# The C-terminus of the oncoprotein TGAT is necessary for plasma membrane association and efficient RhoA-mediated signaling

**DOI:** 10.1186/s12860-018-0155-2

**Published:** 2018-06-07

**Authors:** J. van Unen, D. Botman, T. Yin, Y. I. Wu, M. A. Hink, T. W. J. Gadella, M. Postma, J. Goedhart

**Affiliations:** 1 Swammerdam Institute for Life Sciences, Section of Molecular Cytology, van Leeuwenhoek Centre for Advanced Microscopy, University of Amsterdam, P.O. Box 94215, NL, -1090 GE Amsterdam, The Netherlands; 2 Center for Cell Analysis and Modeling, University of Connecticut Health Center, 400 Farmington Avenue, Farmington, CT 06032-6406 USA

## Abstract

**Background:**

Rho guanine exchange factors (RhoGEFs) control cellular processes such as migration, adhesion and proliferation. Alternative splicing of the RhoGEF Trio produces TGAT. The RhoGEF TGAT is an oncoprotein with constitutive RhoGEF activity. We investigated whether the subcellular location of TGAT is critical for its RhoGEF activity.

**Methods:**

Since plasma membrane associated RhoGEFs are particularly effective at activating RhoA, plasma membrane localization of TGAT was examined. To this end, we developed a highly sensitive image analysis method to quantitatively measure plasma membrane association. The method requires a cytoplasmic marker and a plasma membrane marker, which are co-imaged with the tagged protein of interest. Linear unmixing is performed to determine the plasma membrane and cytoplasmic component in the fluorescence signal of protein of interest.

**Results:**

The analysis revealed that wild-type TGAT is partially co-localized with the plasma membrane. Strikingly, cysteine TGAT-mutants lacking one or more putative palmitoylation sites in the C-tail, still showed membrane association. In contrast, a truncated variant, lacking the last 15 amino acids, TGAT^Δ15^, lost membrane association. We show that membrane localization of TGAT was responsible for high RhoGEF activity by using a RhoA FRET-sensor and by determining F-actin levels. Mutants of TGAT that still maintained membrane association showed similar activity as wild-type TGAT. In contrast, the activity was abrogated for the cytoplasmic TGAT^Δ15^ variant. Synthetic recruitment of TGAT^Δ15^ to membranes confirmed that TGAT effectively activates RhoA at the plasma membrane.

**Conclusion:**

Together, these results show that membrane association of TGAT is critical for its activity.

**Electronic supplementary material:**

The online version of this article (10.1186/s12860-018-0155-2) contains supplementary material, which is available to authorized users.

## Background

Rho GTPases are a subclass of the Ras superfamily of small GTPases, best known for their regulation of the cytoskeleton in eukaryotes [1, 2]. Through remodeling of the F-actin network they regulate several important cellular processes like cell migration, cell adhesion, proliferation and cell shape [3–5]. Rho GTPases function as molecular switches that cycle between an active GTP-bound form and an inactive GDP-bound form [6]. There are several classes of regulatory proteins that influence Rho GTPase activation cycle. Rho guanine exchange factors (RhoGEFs) activate Rho GTPases by accelerating the exchange of GDP for GTP [7]. Rho GTPase activating or accelerating proteins (RhoGAPs) are responsible for turning Rho GTPases off by promoting the hydrolysis of the bound GTP to GDP [8]. RhoGDIs sequester Rho GTPases in the cytoplasm in their inactive GDP bound state by binding to their prenylated C-tails [9, 10]. Deregulation of the Rho GTPase cycle has been mainly investigated within the context of cancer [11] and metastasis [12], but is also implicated in other pathologies like neurodegeneration [13], hypertension [14] and hemopathies [15].

The RhoGEF TGAT (*trio*-related transforming gene in Adult T-cell leukemia) is formed by alternative splicing of the gene product from the RhoGEF Trio and was first identified as an oncogenic gene product in adult T-cell leukemia cells [16]. TGAT consists of 255 amino acids encoding the second C-terminal RhoA activating Dbl homology (DH) domain of Trio and a unique extra 15 amino acid extension at its C-terminus. Both its RhoGEF activity and the 15 amino acid extension were required for its transforming potential in NIH3T3 cells in vitro and in vivo [16], for the activation of tumorigenic transcription factor NF-κB via the IκB kinase complex [17], and the stimulation of matrix metalloproteinases (MMPs) via the inhibition of RECK [18]. RhoGEFs have been put forward as possible therapeutic targets for the treatment of cancer [19, 20]. TGAT is also considered as a possible therapeutic target for adult T-cell leukemia and several aptamer-derived inhibitors of TGAT were already developed [21]. The 15 amino acid extension at the C-terminus of TGAT is referred to as the C-tail in this manuscript.

Although Rho GTPases have been studied in detail for decades, the spatial aspects of their regulation are only starting to be uncovered [22]. For instance, RhoA is activated near the plasma membrane by p63RhoGEF. This RhoA specific GEF has a DH domain that has about 70% amino acid sequence homology to Trio/TGAT [23] and its plasma membrane association is controlled by palmitoylation [24, 25]. We have previously shown that plasma membrane located RhoGEF activity efficiently activates RhoA [26]. Since (i) TGAT activates RhoA and (ii) the C-tail is required for transforming activity, we hypothesized that the oncogenic potential of TGAT originates from subcellular targeting signals in the C-tail.

Here, we have used fluorescence microscopy techniques to investigate the effect of the C-tail of TGAT on subcellular location and function. A novel co-localization analysis based on confocal microscopy images shows that TGAT partially co-localized with the plasma membrane, whereas a TGAT mutant without the complete C-tail is exclusively located in the cytoplasm. Furthermore, we show that plasma membrane localization is necessary for actin polymerization and the activation of RhoA by TGAT. To confirm the plasma membrane as the subcellular site of action of TGAT GEF activity towards RhoA, we make use of a chemical heterodimerization system to target TGAT to several subcellular locations, and find that TGAT has the potential to activate RhoA on several endomembranes beside the plasma membrane.

## Methods

### Construction of fluorescent protein fusions

EGFP-C1-TGAT was a kind gift from Susanne Schmidt [21]. mCherry-C1-TGAT (https://www.addgene.org/84334/) was obtained by cutting the EGFP-C1-TGAT vector with XhoI and AgeI and replacing the EGFP for mCherry, cut from mCherry-C1 with the same enzymes. Restriction sites and oligonucleotide overhangs are marked in bold in primer sequences.

To obtain mCherry-C1-TGAT^Δ15^, we performed PCR with EGFP-C1-TGAT as template, by amplifying with forward 5’-GCGCGATCACATGGTCCTG-3′ and reverse 5′- TTT**GGTACC**TCAGGCTACGATTTTCCCGTC-3′. TGAT^Δ15^ was ligated into mCherry-C1 by cutting the vector and PCR product with KpnI and HindIII.

To obtain EGFP-C1-TGAT^C242S^, we performed a mutagenesis PCR with EGFP-C1-TGAT as template, by amplifying with forward 5’-GCGCGATCACATGGTCCTG-3′ and reverse 5’-CTCTTTGAACCGATGGCTCAGGGCTACGATTTTCC-3′. mCherry-C1-TGAT^C242S^ was obtained by cutting the EGFP-C1-TGAT^C242S^ vector with HindIII and AgeI and replacing the EGFP for mCherry, cut from mCherry-C1 with the same enzymes.

To obtain mCherry-C1-TGAT^C253S^ and mCherry-C1-TGAT^C242S, C253S^ (https://www.addgene.org/84336/), we performed a PCR with EGFP-C1-TGAT and EGFP-C1-TGAT^C242S^ as template, respectively, by amplifying with forward 5’-AGGTCTATATAAGCAGAGC-3′ and reverse 5’-TT**GGTACC**TCAAAACCAACTAATTTCACGAAAAGTCTCTTTG-3′. TGAT^C253S^ and TGAT^C242S, C253S^ were ligated into mCherry-C1 by cutting the vector and PCR products with Acc651 and HindIII.

CFP and YFP color variants were obtained by cutting mCherry-C1-TGAT, mCherry-C1-TGAT^Δ15^, mCherry-C1-TGAT^C242S^, mCherry-C1-TGAT^C253S^ and mCherry-C1-TGAT^C242S, C253S^ with AgeI and HindIII and replacing the mCherry with mTurquoise1 or mVenus, cut from mTurquoise1-C1 and mVenus-C1 with the same enzymes.

For the rapamycin experiments, mCherry-FKBP12-C1-TGAT^Δ15^ was created by cutting mCherry-C1-TGAT^Δ15^ with AgeI and HindIII and replacing mCherry with FKBP12-mCherry, cut from FKBP12-mCherry-C1 [26] with the same enzymes.

A membrane targeting sequence (derived from amino acid residue 1–10 of Lck; MGCVCSSNPE) was constructed by annealing [27] two oligonucleotide linkers, 5′-ctagccaccatgggctgcgtgtgcagcagcaaccccgagcta-3′ and 5′-ccggtagctcggggttgctgctgcacacgcagcccatggtgg-3′, with sticky overhangs and inserting it into an mVenus-C1 plasmid cut with NheI and AgeI, resulting in Lck-mVenus (https://www.addgene.org/84337/). Lck-mTurquoise2 (https://www.addgene.org/98822/) was obtained by exchanging mVenus for mTurquoise2 in the Lck-mVenus plasmid by cutting with AgeI and BsrGI.

The Lck-FRB-ECFP(W66A) was a kind gift from M. Putyrski [28]. FRB-ECFP(W66A)-Giantin, ECFP(W66A)-FRB-MoA, mVenus-MKL2 and the DORA RhoA sensors were previously described [26]. In order to obtain mTurquoise2-CAAX(RhoA), two oligonucleotides were annealed as previously described [27]. Annealing forward 5’-**GTAC**aagctgcaagctagacgtgggaagaaaaaatctgggtgccttgtcttgtga**G**-3′ and reverse 5′- **GATC**ctcacaagacaaggcacccagattttttcttcccacgtctagcttgcag**CTT**-3′ oligonucleotides yielded the coding sequence for the last 15 amino acids of the C-terminus of RhoA (LQARRGKKKSGCLVL*) with overhangs (in capitals) on both sides, compatible with BsrGI and BamHI restriction sites. The double stranded linker was ligated into a C1-mTurquoise2 vector cut with BsrGI and BamHI, resulting in mTurquoise2-CAAX(RhoA). FRB-ECFP-CAAX(RhoA) was obtained by ligating the CAAX(RhoA) fragment, cut from mTurquoise2-CAAX(RhoA), into FRB-ECFP(W66A)-Giantin cut with the same enzymes. mRFP-RhoGDI was a kind gift from Martin A. Schwartz [29]. Plasmids constructed in this study will be made available through Addgene: http://www.addgene.org/Dorus_Gadella/.

### Cell Culture & Sample Preparation

HeLa cells (American Tissue Culture Collection: Manassas, VA, USA) were cultured using Dulbecco’s Modified Eagle Medium (DMEM) supplied with Glutamax, 10% FBS, Penicillin (100 U/ml) and Streptomycin (100 μg/ml). Cell culture, transfection and live cell microscopy conditions were previously described [26]. Treatment with 2-bromopalmitate was performed overnight (~ 16 h) at a concentration of 30–60 μM.

### Widefield microscopy

Static and dynamic ratiometric FRET measurements in HeLa cells were performed using a wide-field fluorescence microscope (Axiovert 200 M; Carl Zeiss GmbH) kept at 37 °C, equipped with an oil-immersion objective (Plan-Neo- fluor 40×/1.30; Carl Zeiss GmbH) and a xenon arc lamp with monochromator (Cairn Research, Faversham, Kent, UK). Images were recorded with a cooled charged-coupled device camera (Coolsnap HQ, Roper Scientific, Tucson, AZ, USA). Typical exposure times ranged from 50 ms to 200 ms, and camera binning was set to 4 × 4. Fluorophores were excited with 420 nm light (slit width 30 nm) and reflected onto the sample by a 455DCLP dichroic mirror, CFP emission was detected with a BP470/30 filter, and YFP emission was detected with a BP535/30 filter by rotating the filter wheel. All acquisitions were corrected for background signal and bleedthrough of CFP emission in the YFP channel (around 55% of the intensity measured in the CFP channel). In dynamic experiments, cells were stimulated with 100 nM Rapamycin at the indicated time points (LC Laboratories, Woburn, USA).

In the actin staining experiment, DAPI was excited with 420 nm light (slit width 30 nm) and reflected onto the sample by a 455DCLP dichroic mirror and emission was detected with a BP470/30 filter, YFP was excited with 500 nm light (slit width 30 nm) and reflected onto the sample by a 515DCXR dichroic mirror and emission was detected with a BP535/30 filter. RFP was excited with 570 nm light (slit width 10 nm) and reflected onto the sample by a 585CXR dichroic mirror and emission of RFP was detected with a BP620/60 filter.

### Confocal microscopy

Experiments were performed using a Nikon A1 confocal microscope equipped with a 60× oil immersion objective (Plan Apochromat VC, NA 1.4). For the co-localization experiments the pinhole size was set to 1 Airy unit (< 0.8 μm) and images with 1.5× zoom of 1024 × 1024 pixels were acquired. For the MKL2 translocation experiments, the pinhole size was set to 1 Airy unit (< 0.8 μm) and images were acquired with 1× zoom using tile scans, resulting in images of 4660 × 4660 pixels. Samples were excited with 447 nm, 514 nm and a 561 nm laser line, and reflected onto the sample by a 457/514/561 dichroic mirror. CFP emission was filtered through a BP482/35 emission filter; YFP emission was filtered through a BP540/30 emission filter; RFP emission was filtered through a BP595/50 emission filter. All acquisitions were corrected background signal. To avoid bleed-through, images were acquired with sequential line scanning modus.

### Fluorescence correlation spectroscopy

Cells were transfected with 100 ng of mTurquoise1-TGAT, mTurquoise1- TGAT^C242S, C253S^ and mTurquoise1-TGAT^Δ15^ or mTurquoise1 as control. FCS data was acquired at an Olympus FV1000 confocal microscope equipped with a Picoharp TCSPC module (Picoquant, Germany). Sample were mounted on the table and illuminated with a pulsed 440 nm Picoquant diode laser (20 MHz, 0.6 kW^.^cm^− 2^) using an Olympus UPLS Apo 60× water NA1.2 objective lens. The fluorescence signal was detected for 60–120 s in confocal mode with the pinhole diameter set at 130 μm. The fluorescence passed a 440 dichroic mirror, was filtered by a 460–500 nm emission filter and detected by an avalanche photodiode (MPD). Correlation curves were generated in FFS Dataprocessor (v2.3 SSTC, Belarus) and fitted using a triplet state-diffusion model [30]. Since the TGAT fusion proteins can be present as free cytoplasmic protein and bound to the membrane, two diffusion times were included in the fitting model. The diffusion times were globally linked over the various measurements, the volume shape factor was fixed to the value obtained from the calibration sample and the triplet time was restricted between 0 and 50 μs. To compare the diffusion times between the various measurement days the values were converted into diffusion coefficients (D) [30], taking into account the slightly variable sizes of the detection volume from day to day, calibrated by the mTq1 sample (*D* = 90 μm^2^.s^− 1^ in PBS). The average diffusion coefficient was calculated taking into account the fractions and values of the two retrieved diffusion coefficients.

### Actin staining

HeLa cells transfected with different TGAT constructs were washed with phosphate-buffered saline solution (PBS) and fixed with 4% formaldehyde for 20 min. After washing with PBS, cells were permeabilized with PBS containing 0.2% Triton X-100. After a second wash step with PBS and blocking of non-specific binding by 1% BSA in PBS for 10 min, cells were stained with 0.1 μM TRITC-phalloidin (Sigma-Aldrich) and 0.1 μg/ml DAPI. After washing with PBS, cells were mounted in mowiol based mounting medium (10% (*w*/*v*) Mowiol 4–88 (cat# 81381, Sigma-Aldrich), 25% (*v*/v) glycerol, 100 mM Tris/HCl (pH 8.5)), and fluorescence images were attained using a widefield fluorescence microscope (Axiovert 200 M; Carl Zeiss GmbH).

### Image analysis

ImageJ (National Institute of Health) was used to analyze the raw microscopy images. Further processing of the data was done in Excel (Microsoft Office) and graphs and statistics were conducted using Graphpad version 6.0 for Mac, GraphPad Software, La Jolla California USA, www.graphpad.com.

Boxplots in Figs. 3, 4, 5 and 6 were generated online, using the website http://boxplot.tyerslab.com/.

Boxplot center lines represent the median values; box limits indicate the 25th and 75th percentiles; whiskers extend 1.5 times the interquartile range from the 25th and 75th percentiles; data points from individual cells are plotted as dots. The notches reflect the 95% confidence interval (CI) around the median, which can be used for statistical inference by eye. When the notches of one boxplot do not overlap with the notches of another box the difference between the medians is statistically significant (at an alpha level of 0.05).

For the MKL2 transcription factor experiments, MKL2 intensity in cytoplasm and nucleus were measured and their ratio was determined. The static FRET data in Fig. 4 was processed using a custom made MatLab GUI, which was described before [26].

The confocal image analysis of the plasma membrane localization for the different constructs and controls was performed by using a combination of ImageJ and MatLab scripts (MATLAB, The MathWorks, Inc., Natick, Massachusetts, United States). For each construct confocal images were obtained with a CFP, YFP and RFP channel (1024 × 1024 pixels with pixel size 140 nm). A sequential time series of 8 images was recorded, which were subsequently averaged for each channel. The channels were spatially registered based on a shift determined with the Lucas-Kanade method that was performed on the controls. The positive control, i.e. Lck-CFP, Lck-YFP and soluble RFP was used to determine the spatial shift between the CFP and the YFP channel. The negative control, i.e. Lck-CFP, soluble YFP and RFP was used to determine the spatial shift between the RFP and YFP channel. In each image and channel the background was determined and subtracted from the image, all sets from one construct were subsequently stored as an ImageJ tif ‘hyperstack’. In ImageJ lines (10 px wide and ~ 6–10 μm long) were drawn perpendicular on regions with a well-defined cytoplasm-plasma membrane-extracellular transition, whilst carefully switching between channels to avoid inclusion of spurious structures in the line-scan. Lines were drawn in all possible orientations, in order to avoid any possible bias resulting from imperfect registration. For each image the line-scans were stored as a RoiSet. By using a matlab script the RoiSets from ImageJ were imported and line-scans were performed in Matlab using bilinear interpolation. In one set, all the profiles obtained from the line-scans for all channels were aligned and centered on the plasma membrane peak position in the Lck-CFP channel, and the profiles were oriented in the same manner, i.e. cytoplasm on the left hand side. By using bilinear interpolation all the profiles were placed on the same position axis. The average profiles were calculated for all three channels and any residual background observed in the extracellular space (> 2 μm) was subtracted, after which the channels were normalized to unity based on the cytoplasm region ([− 2.5, − 1.5] μm). Because the Lck-CFP channel also contained a cytoplasmic localization, the cytoplasmic component was subtracted in order to obtain a more accurate plasma membrane component. This corrected Lck-CFP (*f*_*(CFP-RFP)*_) and the RFP (*f*_*RFP*_) profiles were subsequently used to linearly unmix the profile from the YFP channel (*f*_*YFP*_), using constrained linear regression (lqlin) within the region [− 2.25, 2.25] μm. Hence, *f*_*YFP*_ = *a*_*CP*_ *f*_*RFP*_ + *a*_*PM*_ *f*_*(CFP-RFP)*_, where the coefficients *a*_*CP*_ and *a*_*PM*_ were constrained to positive values and correspond to the cytoplasmic and plasma membrane component respectively. All profiles were realigned and centered on the unmixed PM component. The residual peak in the negative control condition (3 ± 1%) is likely to be caused by the difference in the points spread function (PSF) between YFP and RFP. The PSF of RFP is slightly wider than that of YFP, which has a smoothing effect on the mCherry profile. This is also consistent with the shape of the profiles in Fig. 3b and the residuals (not shown). In order to estimate the confidence intervals on the unmixed CP and PM profiles a bootstrap was performed. Random sets (*n* = 1000) were drawn from the original set with replacement, and the same normalization and unmixing was performed on these sets, after which a 95% confidence interval was calculated based on the standard error of the mean.

## Results

### The C-tail of TGAT affects localization

We have previously shown that the DH-domain of p63RhoGEF has relatively low constitutive activity towards RhoA and that the activity is strongly enhanced when the DH domain is recruited to the plasma membrane [26]. The DH domain of p63RhoGEF is highly homologous to the second DH domain from Trio, from which the splice variant TGAT is derived (Additional file 1: Fig. S1). The main difference between TGAT and the DH domain from p63RhoGEF is the C-tail of TGAT, which essential for the oncogenic activity of TGAT [16]. Therefore, we hypothesized that the C-tail plays a role in membrane association of TGAT. In order to investigate the subcellular localization of TGAT *in cyto*, we constructed and visualized fusions of TGAT with a fluorescent protein attached to its N-terminus.

**Fig. 1:**
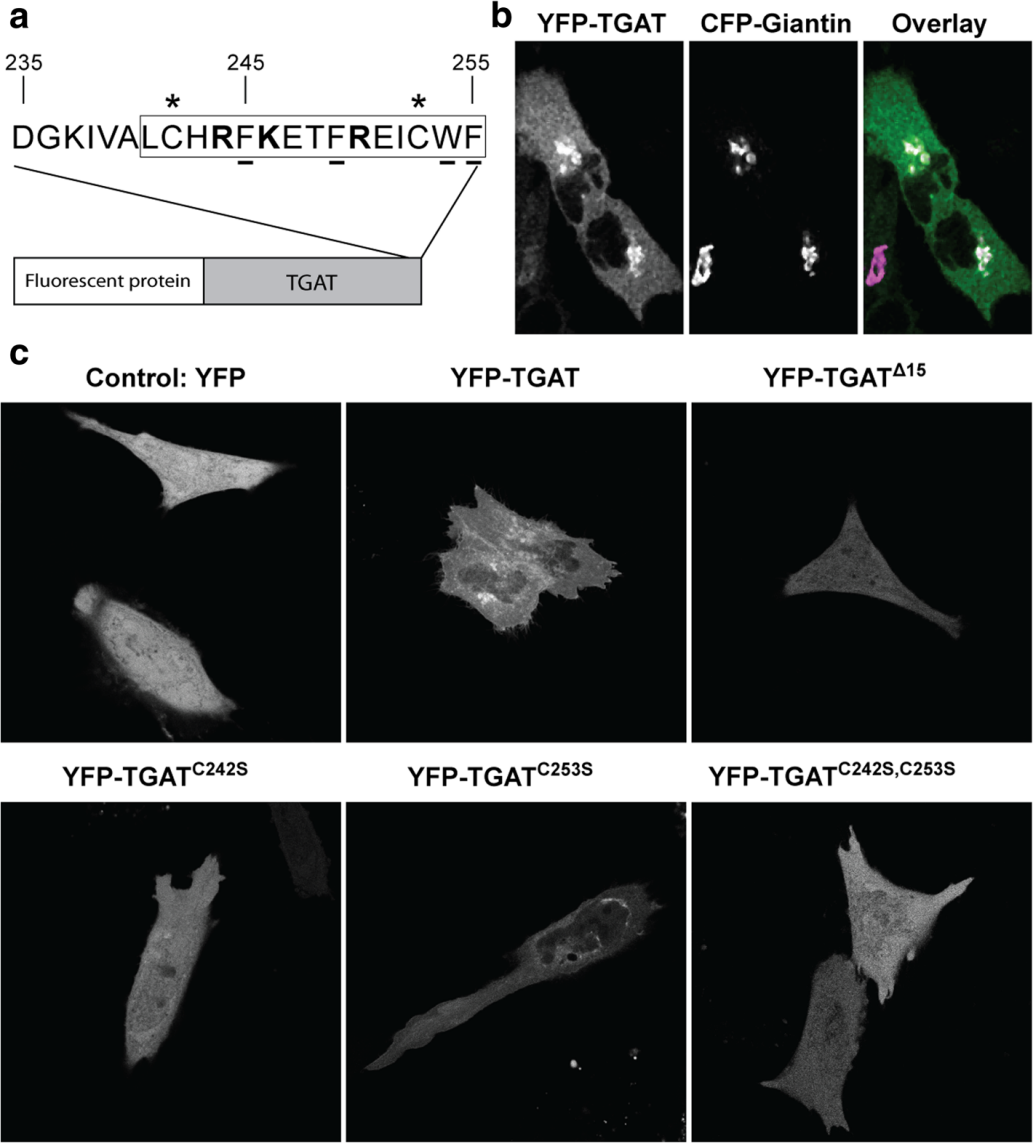
Subcellular localization of TGAT variants and quantification of their plasma membrane localization. **a** Schematic overview of TGAT fused to a fluorescent protein at its N-terminus. The blowout shows the last 20 amino acids of the C-tail of TGAT, with the last 15 amino acids indicated by the rectangle. Positively charged amino acids are shown in bold, amino acids with hydrophobic side-chains are underlined and the two cysteines at position 242 and 253 marked with an asterisk. **b** Confocal microscopy images of HeLa cells co-transfected with YFP-TGAT and the Golgi marker CFP-Giantin. The color overlay was generated by using the TGAT image for the green channel and the Golgi image for the blue and red channel (=magenta). Consequently, colocalized objects appear as white (*right*). **c** Confocal microscopy images of HeLa cells transfected with YFP, YFP-TGAT, YFP-TGAT^Δ15^, YFP-TGAT^C242S^, YFP-TGAT^C253S^, YFP-TGAT^C242S, C253S^. Width of the individual images is 60 μm in (**b**) and 110 μm in (**c**)

The location of YFP-TGAT differed between cells, but we consistently observed that the fluorescence was excluded from the nucleus and increased in a perinuclear compartment (Additional file 2: Fig. S2). Close inspection of the sequence of the last 15 amino acid residues of TGAT reveals two cysteines at position 242 and 253 that are putative palmitoylation sites (Fig. 1a). HeLa cells transfected with YFP-TGAT and a Golgi marker (CFP-Giantin) showed a strong co-localization of TGAT with the Golgi apparatus, as previously observed for proteins that are palmitoylated [31, 32] (Fig. 1b).

To inhibit palmitoylation we use the inhibitor 2-bromopalmitate (2-BP). A fusion protein with the N-terminal peptide sequence from GAP43 is located at the plasma membrane by palmitoylation and served as a control for the efficacy of the drug. Overnight incubation with 2-BP resulted in a complete loss of membrane localization of GAP43, reflecting full inhibition of palmitoylation (Additional file 2: Fig. S2). In the cells treated with 2-BP we observed that TGAT was excluded from the nucleus, showed increased fluorescence near the nucleus in some cells and a more homogeneous localization in the cytoplasm. However, firm conclusions with respect to the effect of 2-BP on the localization cannot be drawn due to cell-to-cell variation.

To prevent lipid modification at the cysteines, we constructed TGAT mutants by replacing the cysteine at amino acid position 242 (TGAT^C242S^) or 253 (TGAT^C253S^) or at both sites (TGAT^C242S, C253S^) by a serine. Confocal images were taken of HeLa cells transfected with a soluble YFP, YFP-TGAT, YFP-TGAT^Δ15^, YFP-TGAT^C242S^, YFP-TGAT^C253S^ or YFP-TGAT^C242S, C253S^ (Fig. 1c). The Golgi apparatus localization of TGAT was clearly diminished in the YFP-TGAT^Δ15^ and YFP-TGAT^C242S, C253S^ mutants, but still present to some extent in the YFP- TGAT^C242S^ and YFP-TGAT^C253S^ mutants. Together, these results support the notion that the C-tail of TGAT determines subcellular localization and that the cysteines may play a role in the affinity for the Golgi.

### A new method to detect plasma membrane co-localization

It was previously shown that positioning the DH domain of p63RhoGEF at the plasma membrane is sufficient to induce constitutive GEF activity towards RhoA and induce actin remodeling [26]. Since TGAT consists of a homologous DH domain, its oncogenic potential might originate from localization at the plasma membrane. Because plasma membrane localization is not immediately apparent from the confocal images of TGAT (Fig. 1b and c), we hypothesized that the fraction of TGAT at the plasma membrane was small compared to the unbound, intracellular pool. In order to quantify the putative plasma membrane localization of TGAT and its mutants, we developed a novel co-localization method to analyze the confocal images of HeLa cells.

The method employs an untagged soluble RFP as marker for the cytoplasm and a lipid-modified CFP (Lck-CFP) as marker for the plasma membrane. The protein of interest with unknown localization is tagged with YFP, and its fluorescence is attributed to either the cytoplasm or the plasma membrane by linear unmixing. We briefly describe the procedure here, a detailed description of the quantification method can be found in the Material and Methods.

**Fig. 2:**
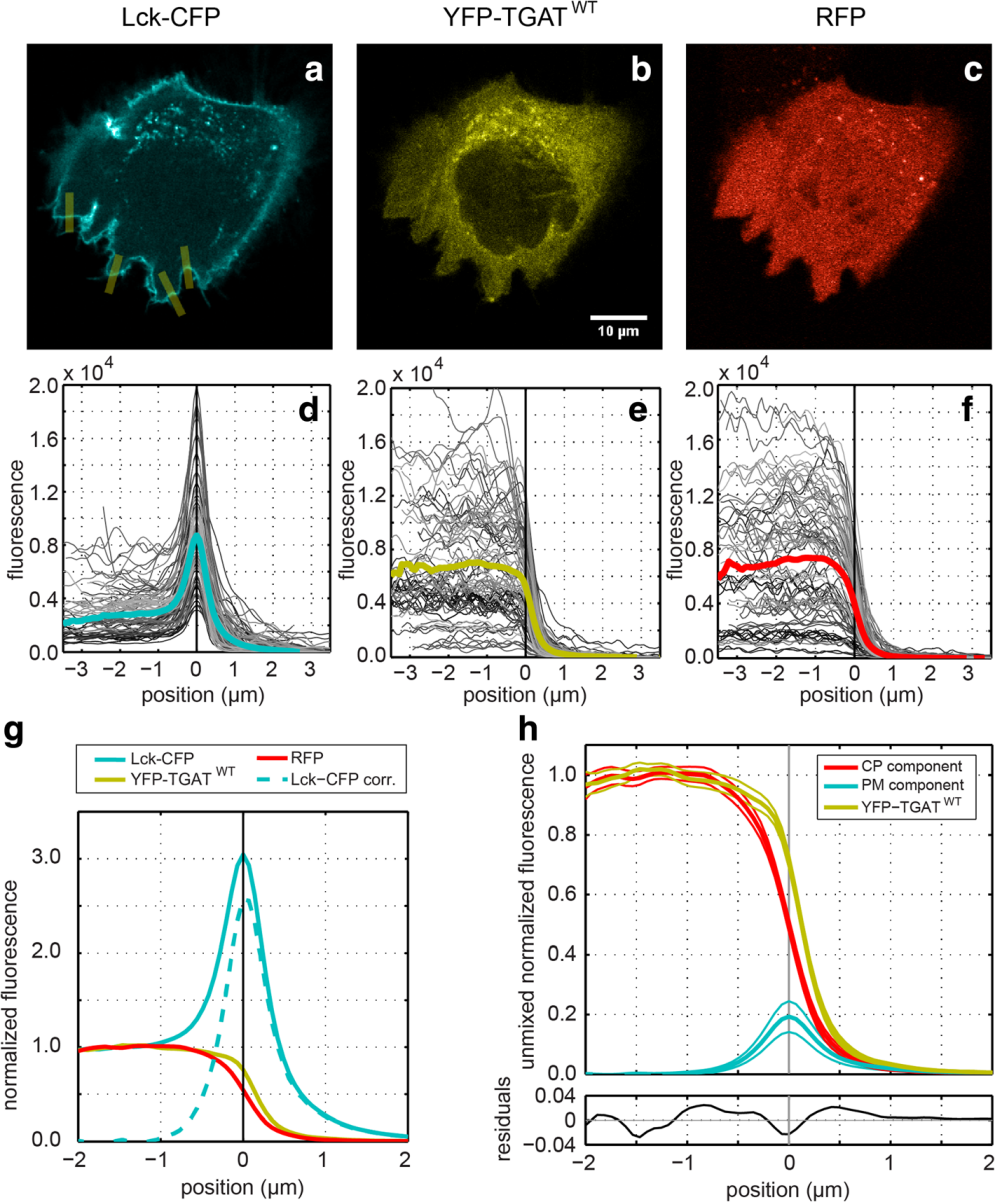
Quantification method for plasma membrane localization. High resolution confocal images (1024 × 1024 px with a pixel size of 140 nm) were taken of HeLa cells transfected with: (**a**) a plasma membrane marker Lck fused to CFP, (**b**) a protein of interest fused to YFP (wild type TGAT in this example) and (**c**) a cytoplasmic marker using a soluble RFP. The CFP channel was registered to the YFP channel based on the spatial shift determined with the positive control. The RFP channel was registered to the YFP channel based on the spatial shift determined with the negative control. Background fluorescence was subtracted from each image prior to processing. Using ImageJ many lines (10 px wide and 6–10 μm long) were carefully drawn perpendicular to the plasma membrane in regions with a well-defined cytoplasm-plasma membrane-extracellular space transition (yellow lines in panel **a**). For each channel line-scans were performed with the same lines using linear interpolation, the profiles were aligned and centered based on the peak in the Lck-CFP channel and placed on the same axis (panel **d**). The other channels were aligned and centered using the same positional shift and axis (panels **e** and **f**). Subsequently the average profiles were calculated (colored lines in panels **d**-**g**), and normalized to unity with respect to the cytoplasm fluorescence level (panel **g**). Because the Lck-CFP marker is also localized in the cytoplasm, the profile was corrected by subtracting the normalized RFP profile (dashed line panel **g**). In order to extract the cytoplasmic (CP) and plasma membrane (PM) component, the YFP profile was unmixed using the normalized RFP profile and the corrected Lck-CFP profile (panel **h**). The 95% confidence intervals (thin solid lines above and below the profiles) were estimated using bootstrapping (See material and Methods for details)

The CFP, YFP and RFP images were spatially registered and background subtracted (Fig. 2a-c). Several perpendicular lines were carefully drawn on regions with a clean cytoplasmic-plasma membrane-extracellular transition in all possible orientations, from which average profiles were obtained using line-scans (normalized to cytoplasm) (Fig. 2d-g). Subsequently, the average profiles were normalized to unity with respect to the cytoplasmic fluorescence level and, because the Lck-CFP marker is also localized in the cytoplasm, the profile was corrected by subtracting the normalized RFP profile (Fig. 2g). The profiles from the YFP channel were subsequently unmixed into a cytoplasmic (CP) and plasma membrane (PM) component using constrained linear regression (Fig. 2h). This procedure allowed for the detection of minute plasma membrane fractions.

### The C-tail is responsible for plasma membrane localization of TGAT

**Fig. 3:**
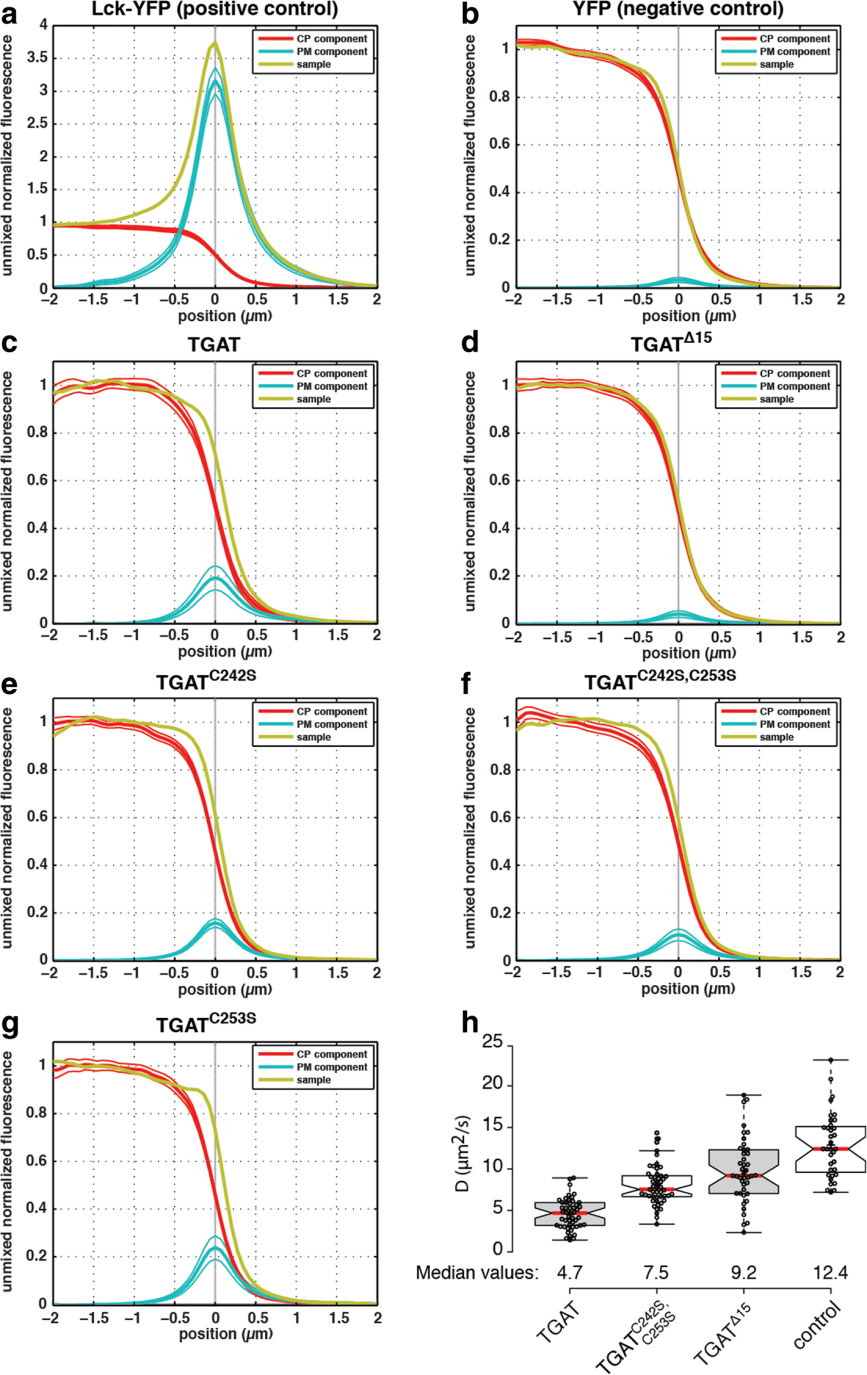
Determination of plasma membrane localization of all TGAT variants. Confocal microscopy was performed on HeLa cells transfected with a plasma membrane marker (Lck-CFP), a cytoplasm marker (soluble RFP) and either (**a**) Lck-YFP (*n* = 67 lines, 10 cells), (**b**) YFP (*n* = 66 lines, 12 cells), (**c**) YFP-TGAT (*n* = 88 lines, 30 cells), (**d**) YFP-TGAT^Δ15^ (*n* = 130 lines, 19 cells), (**e**) YFP-TGAT^C242S^ (*n* = 129 lines, 12 cells), (**f**) YFP-TGAT^C242S, C253S^ (*n* = 112 lines, 12 cells) or (**g**) YFP-TGAT^C253S^ (*n* = 79 lines, 5 cells). Averaged line profiles were obtained using a Matlab script and ImageJ, the profiles were normalized to cytoplasmic levels. Line profiles for the YFP channel (*yellow*) were subsequently unmixed in order to obtain the plasma membrane (*blue*) and cytoplasmic (*red*) component in each construct of interest. The solid thin lines above and below the PM and CP profiles represent the 95% confidence interval obtained from statistical bootstrapping. See Material and Methods for more details. **h** Diffusion coefficients of different TGAT mutants, as determined by fluctuation correlation spectroscopy (FCS). Diffusion coefficients were determined for HeLa cells transfected with CFP-TGAT (*n* = 49), CFP-TGAT^C242S, C253S^ (*n* = 49), CFP-TGAT^Δ15^ (*n* = 42) or free CFP (control, *n* = 35). Boxplot center lines represent the median values (*red*); box limits indicate the 25th and 75th percentiles as determined by R software; whiskers extend 1.5 times the interquartile range from the 25th and 75th percentiles; data points from individual cells are plotted as dots

To examine the plasma membrane localization of TGAT and its C-tail mutants, we employed the novel co-localization analysis. First, we examined the dynamic range of our method by analyzing maximal and minimal plasma membrane association by employing a plasma membrane associated YFP (Lck-YFP) and a soluble YFP respectively. For the membrane bound positive control (Lck-YFP) we determined a PM-localized peak of 316 ± 20% (Fig. 3a), whereas for the cytosolic negative control the PM localized peak was 100-fold lower, 3 ± 1% (Fig. 3b).

Next, we examined the plasma membrane association of TGAT and its C-tail mutants, i.e. YFP-TGAT, YFP-TGAT^Δ15^, YFP-TGAT^C242S^, YFP-TGAT^C253S^ or YFP-TGAT^C242S, C253S^. Wild type TGAT (Fig. 3c) clearly has a detectable PM component of about 19 ± 5%, which is significantly higher than the negative control. This indicates that TGAT is partly localized at the PM, albeit with a more than 10-fold lower level compared to the positive control Lck-YFP. The mutant TGAT^Δ15^, has a PM peak value of 4 ± 1%, which is statistically indistinguishable from the negative control, implying a full cytoplasmic localization (Fig. 3d).

The mutants with the single point mutations, TGAT^C242S^ and TGAT^C253S^ (Fig. 3e and g) exhibit PM peaks of 16 ± 2% and 24 ± 5% respectively. These levels are comparable to TGAT and suggest that these mutants are still localized at the plasma membrane at similar levels as TGAT. The variant with the double point mutation, TGAT^C242S, C253S^ exhibits a PM peak of 11 ± 3%, which is twofold lower than the TGAT level (Fig. 3f), but higher than the negative control at a 95% confidence level.

Thus far, the results suggest that the C-tail confers plasma membrane association. To independently verify this observation, we decided to measure the diffusional mobility of TGAT and its mutants. If wild type TGAT with the intact C-tail or the double mutant, TGAT^C242S, C253S^, bind membranes, they should diffuse slower through the cell than TGAT^Δ15^. To investigate this hypothesis, we employed *in cyto* fluorescence correlation spectroscopy (FCS) to measure and compare the mobility of different TGAT variants. HeLa cells were transfected with CFP-TGAT, CFP-TGAT^C242S, C253S^, CFP-TGAT^Δ15^ or just a soluble CFP and point scanning FCS measurements were performed in cell peripheries. Diffusion times were obtained by fitting the autocorrelation curves (see material and methods for details). From this, diffusion coefficients were calculated by correcting the measured diffusion time with the calibrated detection volume. Both CFP-TGAT (*D* = 4.7 μm^2^/s, 95% CI [4.1–5.3]) and CFP-TGAT^C242S, C253S^ (*D* = 7.5 μm^2^/s, 95% CI [7.0–8.1]) exhibit a lower mobility than CFP-TGAT^Δ15^ (*D* = 9.2 μm^2^/s, 95% CI [8.0–10.5]), although CFP-TGAT is still considerably less mobile than the double cysteine mutant (Fig. 3h). Soluble CFP exhibits the highest mobility (*D* = 12.4 μm^2^/s, 95% CI [11.0–13.9]) through the cell periphery.

Altogether, our results show that the C-tail of TGAT confers plasma membrane association. The double cysteine mutant TGAT^C242S, C253S^ also shows plasma membrane association, suggesting that lipid modification of TGAT by palmitoylation is not necessary for membrane affinity.

### The C-tail of TGAT is necessary for activation of the small GTPase RhoA

We next set out to explore the influence of plasma membrane affinity of TGAT on its functional properties. In order to investigate the possible activation of RhoA by TGAT and its mutants, we transfected HeLa cells with the DORA RhoA biosensor and one of the TGAT variants.

The FRET based biosensor comprises a RhoA-GTP binding domain (PKN), a CFP-YFP FRET pair and RhoA. The PKN does not bind RhoA-GDP and FRET, or the YFP/CFP ratio, is low. When RhoA is active, i.e. bound to GTP, the PKN binds and FRET, or the YFP/CFP ratio, is high.

**Fig. 4:**
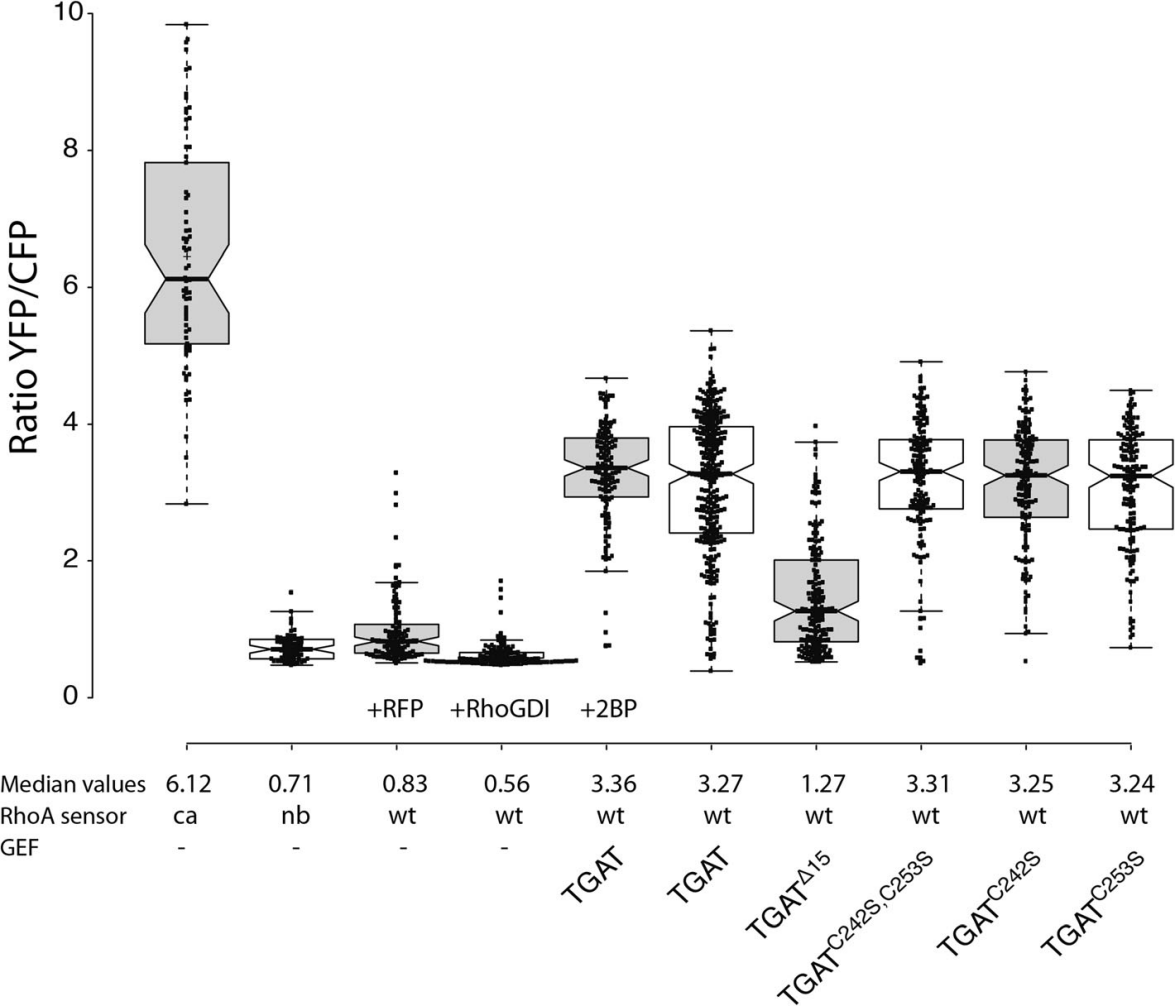
Basal activation of RhoA by the different TGAT mutants. Boxplot showing the median basal YFP/CFP ratio of the DORA RhoA biosensor in HeLa cells. Cells transfected with the constitutive active (ca, *n* = 70) or non-binding (nb, *n* = 62) RhoA biosensor were co-transfected with an empty vector containing only RFP to keep expression levels equal between the different experimental conditions. Wild-type (wt) RhoA biosensor was transfected with an empty vector containing just RFP (control, *n* = 107), RFP-RhoGDI (*n* = 118), RFP-TGAT (+ *2-bromopalmitate*) (*n* = 129), RFP-TGAT (*n* = 291), RFP-TGAT^Δ15^ (*n* = 169), RFP-TGAT^C242S, C253S^ (*n* = 148), RFP-TGAT^C242S^ (*n* = 153) or RFP-TGAT^C253S^ (*n* = 150). Boxplot center lines represent the median values; box limits indicate the 25th and 75th percentiles as determined by R software; whiskers extend 1.5 times the interquartile range from the 25th and 75th percentiles; data points from individual cells are plotted as dots

The YFP/CFP ratio was used to assess the RhoA activation state in each condition (Fig. 4). The minimal and maximal FRET ratios for the DORA RhoA biosensor were estimated from an inactive non-binding (nb) biosensor (nb, 0.71, 95% CI [0.65–0.77]) and a constitutive active (ca) biosensor (ca, 6.12, 95% CI [5.62–6.62]) variant, respectively. The values of the wild-type (wt) DORA RhoA biosensor are expected to fall within this range of YFP/CFP ratios. Control cells transfected with the wild-type biosensor and RFP-RhoGDI show a decreased basal median ratio (0.56, 95% CI [0.54–0.58]) compared to cells transfected with wild-type biosensor and a soluble RFP (0.83, 95% CI [0.75–0.87]), illustrating the preserved regulation of the DORA RhoA biosensor by RhoGDIs. Cells transfected with the sensor and RFP-TGAT^Δ15^ showed much lower basal median YFP/CFP FRET ratio (1.27, 95% CI [1.13–1.42]) when compared to the RFP-TGAT condition (3.27, 95% CI [3.13–3.41]). Cells transfected with the sensor and RFP-TGAT^C242S^ (3.25, 95% CI [3.11–3.39]), RFP-TGAT^C253S^ (3.24, 95% CI [3.07–3.41]) or RFP-TGAT^C242S, C253S^ (3.31, 95% CI [3.18–3.44]) did not show a difference in basal median YFP/CFP FRET ratio compared to the RFP-TGAT condition. Overnight incubation of cells with 2-bromopalmitate (2-BP) was performed to inhibit putative palmitoylation. No effect of 2-BP on the YFP/CFP activity measured in cells with wild-type TGAT was observed (3.36, 95% CI [3.24–3.48]) when compared to the untreated cells (3.27, 95% CI [3.13–3.41]). Taken together, these results show that the plasma membrane localization of TGAT, conferred by the C-tail, results in strong RhoA activation.

### The influence of the C-tail of TGAT on actin polymerization

**Fig. 5:**
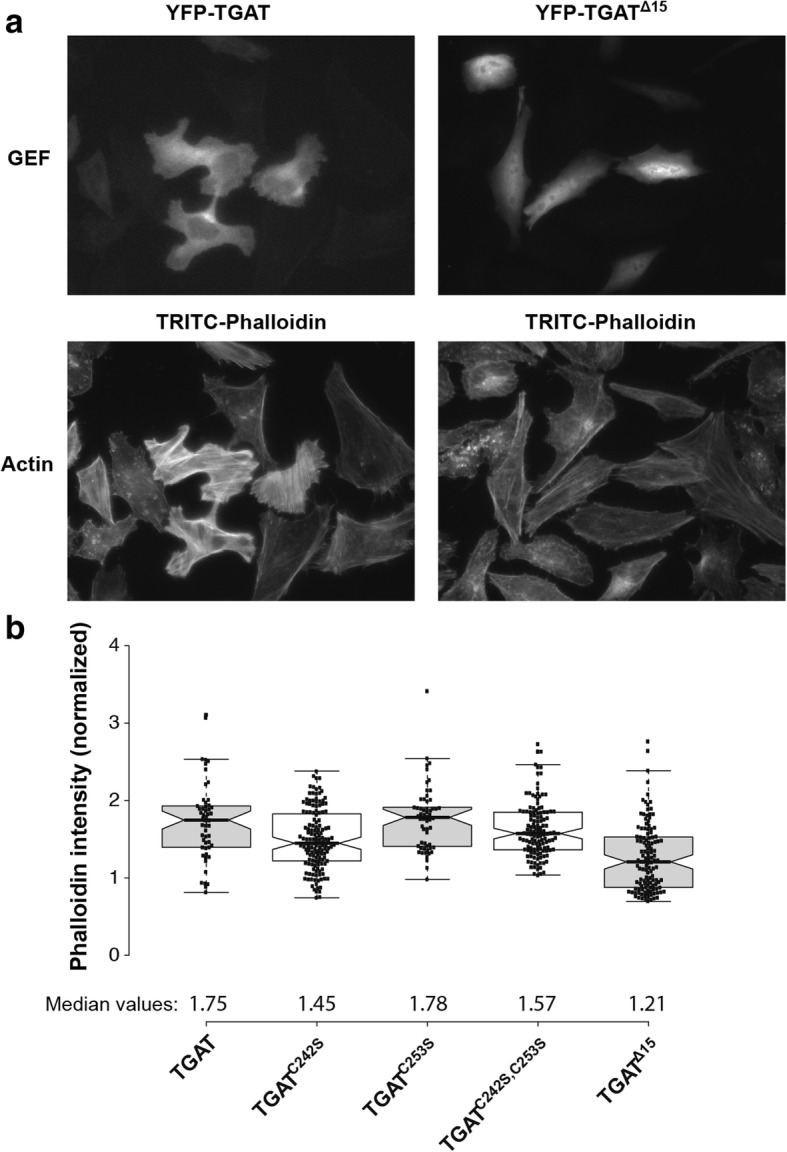
Effect of TGAT mutant expression on actin polymerization. **a** Representative images of HeLa cells transfected with YFP-TGAT or YFP-TGAT^Δ15^ (*top*), stained with TRITC-phalloidin (*bottom*). **b** Quantification of F-actin in HeLa cells transfected with YFP-TGAT (*n* = 53), YFP-TGAT^C242S^ (*n* = 147), YFP-TGAT^C253S^ (*n* = 58), YFP-TGAT^C242S, C253S^ (*n* = 128), YFP-TGAT^Δ15^ (*n* = 126) and stained with DAPI and TRITC-phalloidin, as determined by the fluorescent intensity of the TRITC-phalloidin staining. Actin intensity of transfected cells was normalized to the intensity of untransfected control cells in the same field of view

Previously, we have shown that plasma membrane located RhoGEF activity results in increased actin polymerization, which was not observed for cytoplasmic located RhoGEF activity [26]. In order to investigate the influence of TGAT and its mutants on levels of polymerized actin, HeLa cells were transfected with YFP-TGAT, YFP-TGAT^C242S^, YFP-TGAT^C253S^, YFP-TGAT^C242S, C253S^ or YFP-TGAT^Δ15^. One day after transfection, cells were fixed and stained with an F-actin marker (TRITC-phalloidin) to investigate the influence of the different TGAT mutants on actin polymerization. A clear difference in phalloidin staining intensities was observed between cells transfected with YFP-TGAT and cells transfected with YFP-TGAT^Δ15^ (Fig. 5a). Levels of F-actin were analyzed quantitatively for the different TGAT variants by normalizing the fluorescence intensities of the phalloidin staining of transfected cells to non-transfected control cells within the same field of view (Fig. 5b). These results show that the YFP-TGAT (1.75, 95% CI [1.63–1.87]), YFP-TGAT^C242S^ (1.45, 95% CI [1.37–1.53]), YFP-TGAT^C253S^ (1.78, 95% CI [1.68–1.88]), YFP-TGAT^C242S, C253S^ (1.57, 95% CI [1.50–1.64]) conditions all increase actin polymerization compared to TGAT^Δ15^ (1.21, 95% CI [1.12–1.30]).

**Fig. 6:**
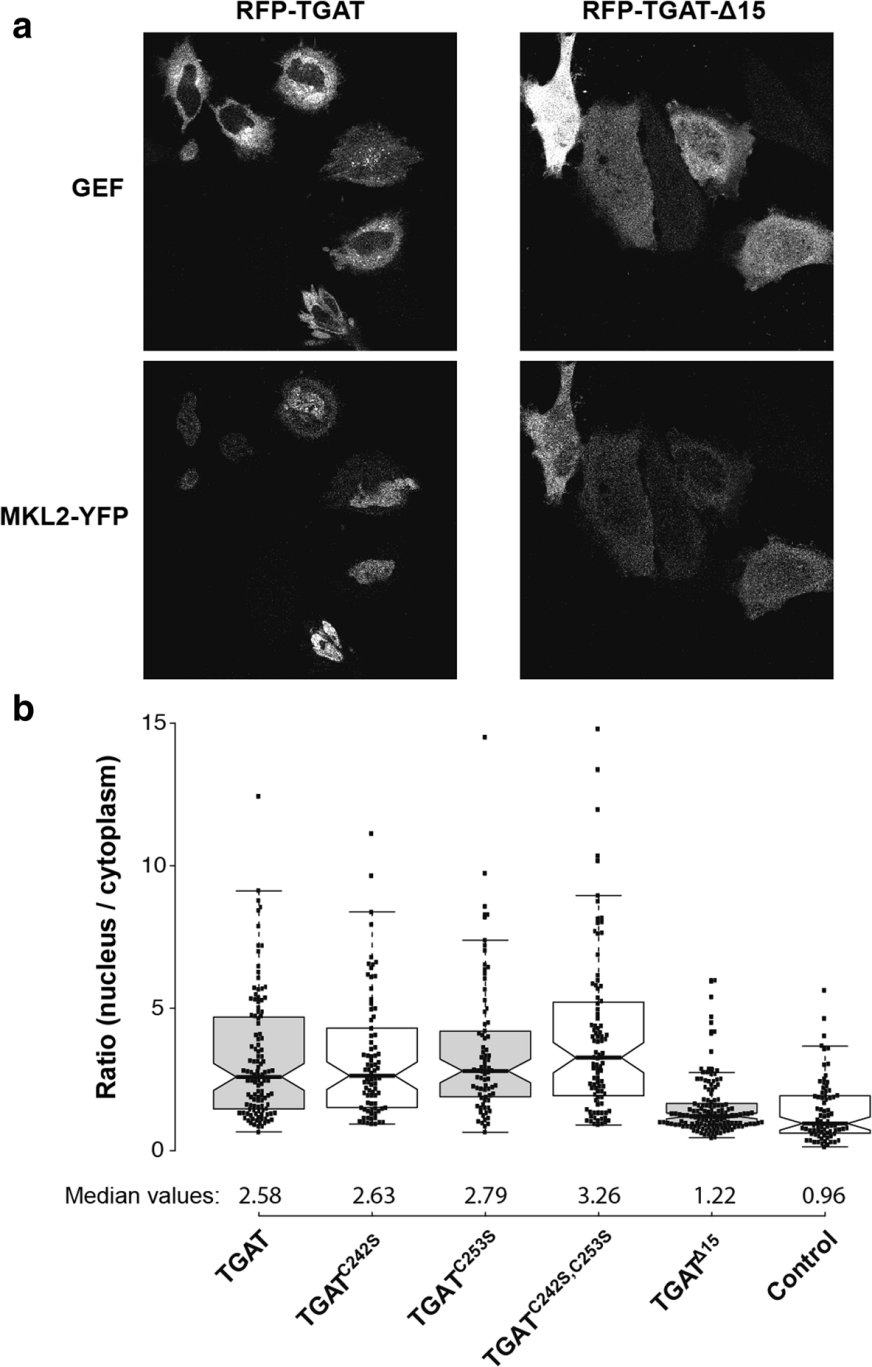
Effect of TGAT mutant expression on nuclear MKL2 translocation.**a** Representative images of HeLa cells co-transfected with YFP-MKL2 (*bottom*) and RFP-TGAT or RFP-TGAT^Δ15^ (*top*). **b** Quantification of the ratio between nuclear and cytoplasmic MKL2-YFP fluorescence in HeLa cells transfected with RFP-TGAT (*n* = 116), RFP-TGAT^C242S^ (*n* = 82), RFP-TGAT^C253S^ (*n* = 77), RFP-TGAT^C242S, C253S^ (*n* = 95), RFP-TGAT^Δ15^ (*n* = 142) or RFP (control, *n* = 77)

Another way to quantify actin polymerization in cells is to determine the subcellular localization of the transcription factor Megakaryoblastic Leukemia 2 (MKL2). MKL2 can bind three G-actin molecules through its RPEL motifs, which are released during actin polymerization in cells [33, 34]. Upon release of the bound G-actin, MKL2 translocates to the nucleus, making it a bona fide sensor for the G-actin / F-actin status in cells [35]. To investigate the influence of the different TGAT variants on the subcellular localization of MKL2, HeLa cells were transfected with MKL2-YFP and the different TGAT variants. Most cells transfected with RFP-TGAT showed a nuclear localization of YFP-MKL2, while YFP-MKL2 was almost always located in the cytoplasm of cells transfected with RFP-TGAT^Δ15^ (Fig. 6a). In order to quantify the subcellular localization state of MKL2, the ratio between YFP-MKL2 fluorescence in the nucleus and the cytoplasm was determined for a large number of cells in all conditions (Fig. 6b). The median ratio for the RFP-TGAT^Δ15^ condition (1.22, 95% CI [1.12–1.32]) was only slightly higher then the median ratio for the control condition (0.96, 95% CI [0.72–1.20]) with only a soluble RFP transfected. In contrast, the median ratios for the RFP-TGAT (2.58, 95% CI [2.10–3.06]), RFP-TGAT^C242S^ (2.63, 95% CI [2.14–3.12]), RFP-TGAT^C253S^ (2.79, 95% CI [2.37–3.21]), RFP-TGAT^C242S, C253S^ (3.26, 95% CI [2.73–3.79]) were all considerably higher than the control condition (0.96, 95% CI [0.72–1.20]) and the RFP-TGAT^Δ15^ condition (1.22, 95% CI [1.12–1.32]).

From the results of the actin staining and the MKL2 localization, we conclude that TGAT and its cysteine mutants increase the amount of F-actin in cells. In contrast, TGAT^Δ15^ shows hardly any effect on actin polymerization compared to wild-type TGAT. These results are in agreement with the RhoA activity that we measured, and point towards the critical importance of plasma membrane localization for the function of TGAT.

### TGAT activates RhoA at the plasma membrane and mitochondria

In order to investigate our hypothesis that plasma membrane localization of TGAT results in the activation of RhoA, we decided to use a previously described [26, 36] chemical dimerization system based on rapamycin to gain spatiotemporal control over the subcellular location of TGAT in single living cells. We fused TGAT^Δ15^ to FKBP12 and used FRB fused to several subcellular targeting sequences. RhoA activation in single cells was measured over time with the DORA RhoA FRET sensor, before and after targeting FKBP12-TGAT^Δ15^ to Lck-FRB-ECFP(W66A) (plasma membrane), FRB-ECFP(W66A)-CAAX(RhoA) (location of RhoA on endomembranes), ECFP(W66A)-FRB-MoA (mitochondria) or FRB-ECFP(W66A)-Giantin (Golgi apparatus) by adding rapamycin.

**Fig. 7:**
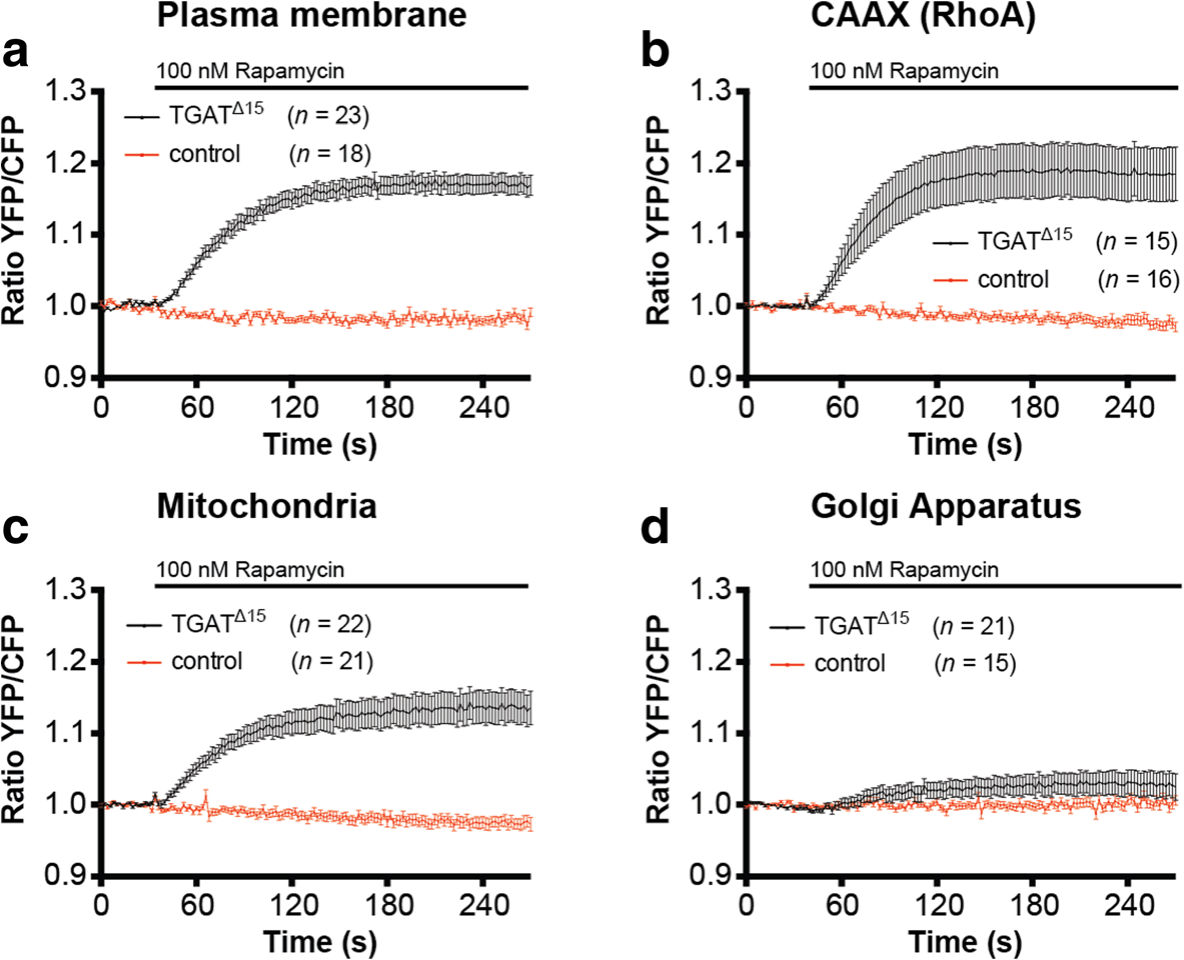
Recruitment of the TGAT^Δ15^ to several subcellular locations results in RhoA activation. **a** Hela cells transfected with the DORA-RhoA biosensor, Lck-FRB-ECFP(W66A) (plasma membrane) and RFP-FKBP12-TGAT^Δ15^ (*n* = 23) or RFP-FKBP12 (control, *n* = 18) were stimulated with Rapamycin (100 nM) at *t* = 32 s. **b** Hela cells transfected with the DORA-RhoA biosensor, FRB-ECFP(W66A)-CAAX(RhoA) and FKBP12-RFP-TGAT^Δ15^ (*n* = 15) or RFP-FKBP12 (control, *n* = 16) were stimulated with Rapamycin (100 nM) at *t* = 32 s. **c** Hela cells transfected with the DORA-RhoA biosensor, MoA-FRB-CFP(W66A) (mitochondria) and RFP- FKBP12-TGAT^Δ15^ (*n* = 22) or RFP-FKBP12 (control, *n* = 21) were stimulated with Rapamycin (100 nM) at *t* = 32 s. **d** Hela cells transfected with the DORA-RhoA biosensor, Giantin-FRB-CFP(W66A) (Golgi apparatus) and RFP- FKBP12-TGAT^Δ15^ (*n* = 21) or RFP-FKBP12 (control, *n* = 15) were stimulated with Rapamycin (100 nM) at *t* = 32 s. Time traces show the average ratio change of YFP/CFP fluorescence (±s.e.m)

Recruiting TGAT^Δ15^ to the plasma membrane (Fig. 7a) or the CAAX-box of RhoA (Fig. 7b) resulted in a fast and sustained increase in RhoA biosensor activation. Interestingly, targeting TGAT^Δ15^ to mitochondria also resulted in a fast and sustained increase in RhoA activation (Fig. 7c), whereas targeting TGAT^Δ15^ to the Golgi apparatus (Fig. 7d) only lead to a minimal response on the RhoA sensor. These results show that the RhoGEF activity of TGAT is strongly enhanced when TGAT is located on membranes.

## Discussion

Despite the well-established observation that the C-tail of TGAT is essential for its oncogenic potential, it has been unclear what underlying molecular mechanism is responsible. Here, we showed that ectopically expressed TGAT is localized to endomembranes, especially the Golgi apparatus. Using a novel quantitative co-localization analysis method for confocal images, we showed that a fraction of wild type TGAT is located at the plasma membrane and that TGAT lacking the C-tail, TGAT^Δ15^, is not. Furthermore, we found that mutating the two cysteines, which are possible palmitoylation sites, did not affect plasma membrane association of TGAT, while it diminished Golgi localization. Functional analysis revealed that TGAT and also its cysteine mutants are capable of activating RhoA, resulting in increased levels of polymerized actin. In contrast, TGAT devoid of the C-tail, TGAT^Δ15^, was severely impaired in the activation of RhoA, actin polymerization or translocation of the transcription factor MKL2 to the nucleus. Synthetic recruitment of TGAT^Δ15^ from the cytoplasm to the plasma membrane, but not the Golgi apparatus, resulted in an increase of GEF activity towards RhoA. Altogether our findings are in line with a model in which the oncogenic activity of TGAT originates from constitutive RhoGEF activity at the plasma membrane.

It was previously shown that plasma membrane localization and function of the Rho GTPase Chp is critically dependent on basic and hydrophobic residues in its C-tail, rather than palmitoylation or prenylation [37]. Although the effects of palmitoylation, prenylation and basic or hydrophobic amino acid stretches on endomembrane affinity of proteins have been extensively studied [38–40], it is still unclear how lipidation exactly influences subcellular location and membrane affinity. Whether increase in post-translational lipidation modifications simply provide cumulative gradual increases to endomembrane and plasma membrane affinity, or that specific lipidation motif exist to target proteins to different subcellular endomembrane locations, is still unclear.

We found that the mobility of the double mutant was higher than wild-type TGAT, which reflects a lower sampling rate of endomembranes due to reduced membrane affinity. Future site mutagenesis studies targeting the basic and hydrophobic residues in the C-tail could possibly shed more light on whether plasma membrane affinity is specifically involved in its oncogenic potential. Another option would be that the C-tail of TGAT contains unknown motifs for protein-protein interactions or targeting to scaffolds. In any case, our results imply that interfering with palmitoylation is not a viable strategy to reduce oncogenic activity of TGAT towards RhoA. This is in contrast to other GTPases like oncogenic RAS, where it has been postulated that interfering with the depalmitoylation machinery might provide therapeutic benefits by mislocalizing RAS activity [41].

The observation that TGAT can activate RhoA when targeted to mitochondrial sites is unexpected. It was previously shown that the DH domain of p63RhoGEF, which shares 70% sequence identity with the DH domain of TGAT at the protein level, does not activate RhoA at mitochondria in a similar assay [26]. Currently, we do not have an explanation for the increased RhoGEF activity that is observed by mitochondrial localized TGAT-DH.

## Conclusion

One striking outcome of our study is that localization of only a minor fraction of protein at membranes is sufficient to cause substantial changes in cell physiology. Importantly, this membrane-localized fraction is almost undetected when employing confocal microscopy under optimal conditions. Only by virtue of a new unmixing-based image analysis strategy, the membrane association can be robustly detected. This method is generally applicable and should be of interest to studies where membrane association is hardly or not visible. Of note, combining the analysis method with high resolution imaging strategies (e.g. SIM or other super resolution techniques) will further lower the limit of detection for membrane association.

In summary, our results highlight a role for the C-terminal 15 amino acids in the membrane association of TGAT and the subsequent activation of RhoA and actin polymerization by TGAT. This study provides a framework to further investigate the exact origin of the oncogenic potential in the C-tail from the RhoGEF TGAT.

## Additional file

Additional file 1 Figure S1 Sequence alignment of TGAT and p63RhoGEF. Protein sequence alignment of the DH domain of human p63RhoGEF and the complete protein sequence TGAT. (PNG 100 kb)

Additional file 2 Figure S2 The localization of YFP-TGAT is hardly affected by the treatment with 2-BP. Confocal images of cells expressing YFP-TGAT and GAP43-CFP. The GAP43 fusion is used to monitor the palmitoylation status of proteins in cells. In untreated cells GAP43 is located at the plasma membrane due to palmitoylation. Overnight treatment with 60 μM 2-BP inhibits palmitoylation of GAP43, which is reflected by the cytoplasmic localization. In contrast to GAP43, the localization of YFP-TGAT is hardly affected by the treatment with 2-BP. (PNG 963 kb)

## Abbreviations

2-BP: 2-bromopalmitate
CFP: Cyan fluorescent protein
CP: Cytoplasm
DH: Dbl homology
MKL2: Megakaryoblastic Leukemia 2
PM: Plasma membrane
RFP: Red fluorescent protein
RhoGEF: Rho guanine exchange factor
TGAT: Trio-related transforming gene in Adult T-cell leukemia
YFP: Yellow fluorescent protein
