# Supplementary figures and images for: The C-terminus of the oncoprotein TGAT is necessary for plasma membrane association and efficient RhoA-mediated signaling
Source: BMC Cell Biol. 2018 Jun 7;19:6. doi: 10.1186/s12860-018-0155-2 (PMC5992656; doi:10.1186/s12860-018-0155-2)

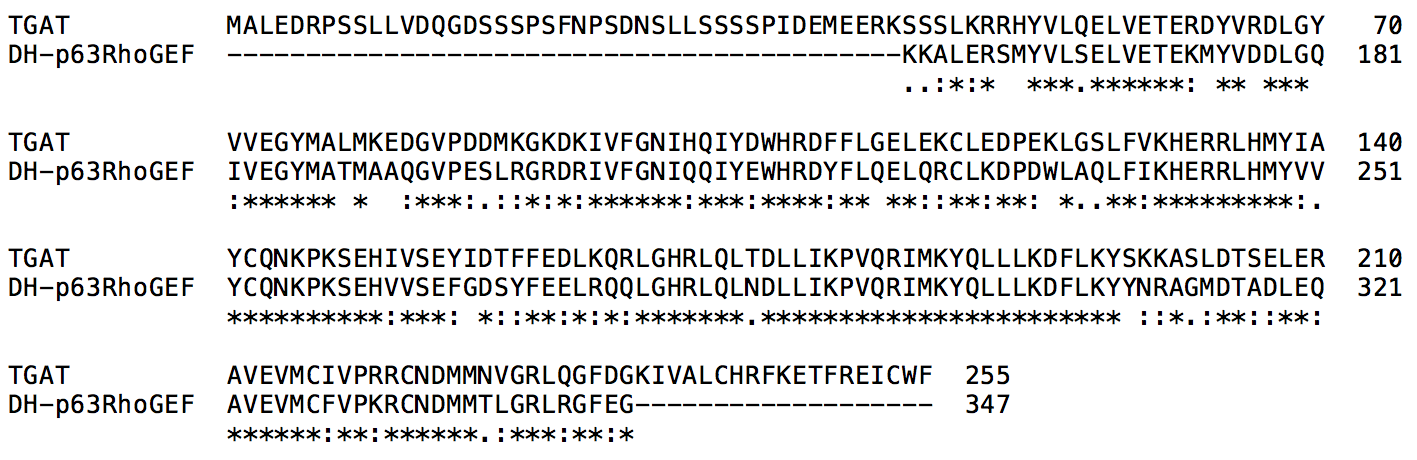

Supplement: Supplementary file 1 — Figure S1 Sequence alignment of TGAT and p63RhoGEF. Protein sequence alignment of the DH domain of human p63RhoGEF and the complete protein sequence TGAT. (PNG 100 kb) [file 12860_2018_155_MOESM1_ESM.png]

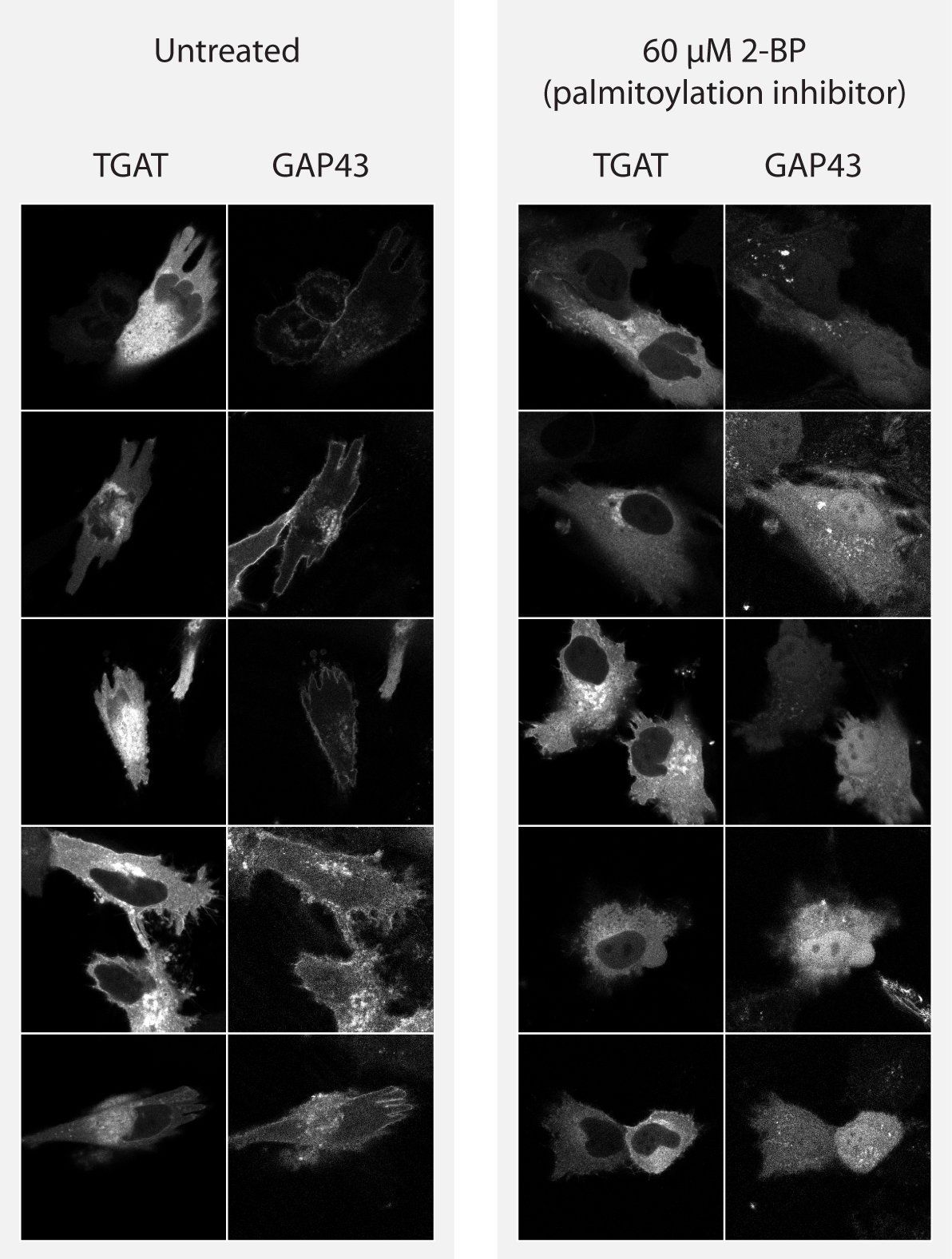

Supplement: Supplementary file 2 — Figure S2 The localization of YFP-TGAT is hardly affected by the treatment with 2-BP. Confocal images of cells expressing YFP-TGAT and GAP43-CFP. The GAP43 fusion is used to monitor the palmitoylation status of proteins in cells. In untreated cells GAP43 is located at the plasma membrane due to palmitoylation. Overnight treatment with 60 μM 2-BP inhibits palmitoylation of GAP43, which is reflected by the cytoplasmic localization. In contrast to GAP43, the localization of YFP-TGAT is hardly affected by the treatment with 2-BP. (PNG 963 kb) [file 12860_2018_155_MOESM2_ESM.png]
